# The Formation and Evolution of the Japan Primary Care Association: A 15‐Year Retrospective

**DOI:** 10.1002/jgf2.70070

**Published:** 2025-10-08

**Authors:** Shinji Matsumura, Masaki Amenomori, Tesshu Kusaba

**Affiliations:** ^1^ Matsumura Clinic Tokyo Japan; ^2^ Yuge Medical Clinic/Shiga Center for Family Medicine Ryuo Shiga Japan; ^3^ Hokkaido Centre for Family Medicine Sapporo Hokkaido Japan

**Keywords:** academic society, family medicine, general practitioner, history, Japan, Japan Primary Care Association, primary care

## Abstract

The Japan Primary Care Association (JPCA) was established in 2010 through the merger of three academic societies: the Japanese Medical Society of Primary Care, the Japanese Academy of Family Medicine, and the Japanese Society of General Medicine. This article reviews the historical development of these predecessor organizations, the context and process of their unification, and the evolution of JPCA. Over the past 15 years, JPCA has promoted clinical practice, education, and research while enhancing inter‐professional collaboration and certification systems for physicians, nurses, and pharmacists. Its proactive response to disasters, involvement during the COVID‐19 pandemic, and participation in healthcare policies reflect its dedication to addressing Japan's changing healthcare landscape. As the leading academic organization for primary care in Japan, JPCA continues to foster integrated community‐based care and aims to strengthen its presence both nationally and internationally in the advancing field of primary care.

## 1 Introduction

In April 2010, the Japanese Medical Society of Primary Care, the Japanese Academy of Family Medicine, and the Japanese Society of General Medicine merged to form the Japan Primary Care Association (JPCA) [1]. This merger marked a significant milestone in the advancement of primary care in Japan. Since its establishment, the JPCA has been at the forefront of shaping the primary care field as the representative academic organization in Japan [2].

As medical care continues to evolve with demographic shifts and a complex globalized environment, the JPCA will continue to guide primary care. The Year 2025 marks the 15th anniversary of the association's founding, a testament to its enduring relevance.

This article examines the history of the JPCA by tracing the origins and evolution of its three predecessor societies, the circumstances surrounding their mergers, and JPCA's key activities since its establishment.

## 2 The Institutional Foundations of Primary Care in Japan: From Clinical Practice to Academic Organization

After World War II in 1945, postwar reconstruction efforts accelerated worldwide, including the re‐establishment of healthcare systems. Since then, Japan's healthcare delivery system and medical education have developed rapidly [3]. However, as the system took shape, it became increasingly centered around higher‐level medical institutions responsible for training healthcare professionals, leading to a hospital‐centered model of medical care [4]. Amidst these developments, efforts were made to explore the uniqueness and universality of primary care from a scientific perspective.

In this context, Tomojiro Nagai, a general practitioner in Mitaka City, Tokyo, published two articles in *Igaku no Ayumi* and the *Japan Medical Association Journal*, titled “Creating Journal Space for Practicing Clinicians” [5] and “Let's Nurture Medicine in the Early Stages of Illness,” [6] respectively. In these publications, he publicly emphasized the expertise of family doctors, advocated for the establishment of academic societies and journals dedicated to their practice, and proposed the creation of a dedicated academic organization for general practitioners [7]. Responding to his call, four physicians took the initiative to form such an organization, leading to the inaugural meeting of the Association of Medical Practitioners (*Jicchi ika no tame no kai*) in Tokyo in February 1963 [8]. Thereafter, the association continued to hold monthly meetings, expand its membership, and work toward establishing a formal academic society for primary care physicians in Japan. This movement predated the publication of *the Millis Commission report*—a landmark report by the Citizens' Commission on Graduate Medical Education in the United States [9]—by 3 years.

In 1978, the Alma‐Ata Declaration, adopted at an international conference convened by the World Health Organization (WHO) and UNICEF, underscored the global importance of primary health care [10]. This milestone led to the establishment of numerous academic organizations for family doctors and general practitioners worldwide. In Japan, building on the foundation of the Association of Medical Practitioners, the Japanese Medical Society of Primary Care was officially established in 1978 [11]. In 1985, the Japanese Medical Society of Primary Care was recognized as an official member of the World Organization of National Colleges, Academies, and Academic Associations of General Practitioners/Family Physicians (WONCA), the leading academic body for family doctors and general practitioners globally. This affiliation integrated the Japanese Medical Society of Primary Care into the international academic network of primary care professionals [12]. Domestically, the association strengthened its presence by organizing study groups and branch meetings in collaboration with prefectural medical associations, ultimately evolving into a nationwide organization.

## 3 The Founding of Primary Care Departments in Japanese Medical Schools

During the 1980s, the importance of primary care departments within Japanese teaching hospitals increased, leading to the introduction of general and family medicine concepts that originated mainly in Western countries, including the United States and European countries. Consequently, departments dedicated to these areas were established in Japan. This movement began with the establishment of the Department of General Medicine and Education, the first of its kind in Japan, at the Tenri Yorozu Hospital, a teaching hospital in Nara Prefecture, in 1976 [13]. Similar departments followed, including the Department of General Medicine at Kawasaki Medical School in 1980 [14] and the Department of Community Medicine at Jichi Medical University in 1981 [15]. In 1986, the Department of General Medicine at Saga Medical School became the first department established at a public university [16]. Concurrently, physicians working in local communities and remote areas, particularly those affiliated with Jichi Medical University, contributed to redefining the role of primary care physicians in remote communities of Japan. Their continuous efforts led to a gradual reevaluation of primary care as a crucial component of Japan's healthcare system. The expansion of academic departments in family medicine and general practice at university hospitals [17], combined with the grassroots medical activities of local clinicians, has steadily fostered a growing base of physicians in this field [2].

## 4 Ministry‐Led Reforms for Postgraduate Training in Primary Care

In parallel with these developments, the Japanese government also began considering policies that promote the training of primary care specialists. As part of these efforts, the Ministry of Health and Welfare in Japan launched the *Rinsho Kensyu Shidou‐I Kaigai Haken Seido* (Clinical Training Supervisor Overseas Dispatch Program) in 1980, marking the beginning of a structured approach to training supervisors in general and family medicine. The first three participants in this program—Nobutaro Ban, Tomoyuki Kido, and Shunichi Fukuhara—were sent to the United States for clinical training. Many physicians have utilized this system to gain experience in family medicine and general internal medicine abroad, and those who completed the program later became key figures in the development of general and family medicine education in Japan.

In 1985, *Katei‐i ni Kansuru Kondankai* (the Round‐Table Conference on Family Physicians) was established within the Ministry of Health and Welfare in Japan, initiating discussions on introducing a formal family physician system. However, due to resistance from key stakeholders, particularly the Japan Medical Association, these discussions were restricted to articulating the “10 items of Functions of Family Physicians” rather than implementing concrete policies. Nevertheless, the Advisory Panel's final report included a “Proposal for the Training of Physicians to Provide Family Medicine Functions,” which maintained momentum for the development of primary care training [18].

## 5 The Launch of the Japanese Academy of Family Medicine and the Japanese Society of General Medicine

In 1984, a study group on family medicine was established by volunteer doctors from around the country, inspired by family medicine movements in the United States and the United Kingdom. Among those involved in launching the initiative were Masaji Maezawa and Tsukasa Tsuda, who made significant contributions to its early development. The first Family Medicine Seminar (The Katei Igaku Seminar) was held in November of the same year, and the quarterly journal “Family Doctor” was launched [19]. The seminar was later renamed to the Family Medicine Seminar (The Katei Iryogaku Seminar), and the Japanese Academy of Family Medicine was established in November 1986. The Japanese Academy of Family Medicine has held annual academic conferences since 1989. Additionally, it hosts a summer seminar on family medicine for students and residents, which is held regularly. Through this seminar, the Japanese Academy of Family Medicine has gradually expanded its membership base by increasing the number of young participants. In 2002, the society evolved into an academic organization.

In 1989, the Japan General Medicine Liaison Council was established as a liaison organization for general medicine departments that were successively established at clinical training hospitals and university hospitals across the country in the 1980s. The Japan General Medicine Study Group was subsequently established, based on this council, and the first study group meeting was held at the National Tokyo Second Hospital (now the National Hospital Organization Tokyo Medical Center) on February 27, 1993. The Japan General Medicine Study Group also continued to grow, with the number of members affiliated with university hospitals and teaching hospitals increasing as it held academic conferences. In 2000, the society was reorganized as the Japanese Society of General Medicine [20].

## 6 Integration of the Three Academic Societies and the Establishment of the Japan Primary Care Association (2010)

Although these academic organizations were founded under different circumstances, they shared many common areas of interest. Throughout the 1990s, each society independently pursued its activities in clinical practice, education, and research. This landscape began to shift in 2004, with the introduction of a new postgraduate clinical training system in Japan. This new system led to more profound personal and academic exchanges, particularly as discussions on shared primary care education and training challenges progressed, especially in regional areas [21].

A key step in the direction of unification occurred in 2001, with the establishment of *the Primary Care Kyoiku Renraku Kyogikai* (Liaison Council of Primary Care Education), which laid the foundation for collaborative educational efforts [22]. Subsequent joint events strengthened inter‐societal cooperation. These included a joint session at the WONCA Asia‐Pacific Regional Conference (the Kyoto International Conference Center, Kyoto) in May 2005, and a collaborative event at the Academic Conference of the Union of Primary Care‐related Academic Societies (comprising the Japanese Medical Society of Primary Care, the Japanese Academy of Family Medicine, and the Clinical Research Interest Group of the Japanese Society of General Medicine) at the Nagoya Congress Center (Aichi Prefecture). In August 2009, the three societies co‐hosted the Joint Conference of Primary Care‐related Academic Societies at the Kyoto International Conference Center [1, 23, 24, 25]. In the same year, they began collaborating with the Japan Medical Association to develop a standard curriculum for continuing medical education. These initiatives laid the groundwork for formal discussions on merging the three organizations [26].

Despite their shared objectives, the three societies encountered various obstacles to unification, as each had a distinct history and philosophy. However, the recognition that a single unified organization would be more effective than multiple independent entities would ultimately gain traction and accelerate the movement toward integration.

After extensive discussions, the Japanese Medical Society of Primary Care, the Japanese Academy of Family Medicine, and the Japanese Society of General Medicine formally agreed to merge. On April 1, 2010, the Japan Primary Care Association (JPCA) was officially established under its first president, Seiji Maezawa [27]. The newly merged association held its first academic conference at the Tokyo International Forum between 26 and 27 June 2010. This marked a significant step forward in unifying academic efforts to advance general practice, family medicine, and community medicine in Japan, further solidifying the foundation for the country's academic and professional development of primary care.

## 7 Development of Specialist and Certified Physician Systems, and the Launch of the Family Medicine Specialist System

Before the JPCA was established, each academic society independently developed its own certification and specialist training systems. The Japanese Medical Society of Primary Care launched its Certified Physician training program in 1994 and introduced the Objective Structured Clinical Examination (OSCE) into its certification process in 2001. In 2003, the association implemented a dual certification system that distinguished between specialists and certified physicians. The Japanese Academy of Family Medicine first started certifying family medicine specialists in 2009. However, with the formation of the JPCA in 2010, these systems were unified into a two‐tiered specialist framework: the JPCA Certified Family Physicians and the JPCA Board Certified Primary Care Physicians. The first family physician examination under this unified system was held on September 20, 2010, to certify the first fifty‐five Japan Primary Care Association‐Certified Family Physicians. In the same year, the Primary Care‐Certified Physician system was launched, with 21 physicians receiving certification under the JPCA.

In 2013, the Ministry of Health, Labour, and Welfare (MHLW) in Japan published the final report of its Study Group on the Ideal Form of Medical Specialists (*Senmon‐i no Arikata ni Kansuru Kentou‐kai*), which proposed the establishment of a neutral third‐party organization, the Japan Medical Specialty Board (JMSB), to oversee specialist training [28]. This new body was tasked with the standardized evaluation and accreditation of specialist training programs across all fields. The report categorized medical specialties into “basic areas” and “subspecialty areas,” establishing the fundamental principle that all clinicians should obtain certification in an essential area of specialty following their initial medical training. This standardized and mandated later‐stage training represented a significant reform in Japan's medical education system. Under this framework, “general medicine” was officially designated as a basic medical specialty [28].

Following this policy shift, the board‐certified specialists of general medicine “Sogo‐Shinryo‐Senmon‐I” system was officially established in 2018, and in 2021, the first 78 board‐certified specialists of general medicine were certified under this system [29]. Importantly, the JPCA played a significant role in both the introduction and the ongoing management of this system.

## 8 Activities Since the Establishment of the Japan Primary Care Association

Since its establishment, the JPCA has actively engaged in various clinical, educational, and research activities. Numerous committees and project teams have been formed within the association, each pursuing unique initiatives.

Among them, the four activities highlighted below were selected for their significance in shaping the identity, scope, and influence of the JPCA over its 15‐year history. These initiatives represent major developments in disaster response, public health leadership, academic recognition, and inter‐professional collaboration—each reflecting a core dimension of the association's evolution.

### 8.1 Primary Care Assistance Team (PCAT)

On March 11, 2011, shortly after the establishment of the JPCA, the Great East Japan Earthquake—a massive magnitude 9.1 earthquake—struck, just off the coast of Miyagi Prefecture. The JPCA assembled a disaster‐relief team in response. On March 17, just 6 days after the disaster, Toshio Naito of Juntendo University was dispatched to Kesennuma City, Miyagi Prefecture, as the first advance disaster‐relief team member [30]. The JPCA formally organized its disaster‐relief team and established the Primary Care Assistance Team (PCAT), which has since been actively involved in disaster response [31]. The PCAT has provided ongoing support for subsequent disasters, including the 2016 Kumamoto Earthquake [32] and the 2024 Noto Earthquake [33].

In 2024, the JPCA introduced a PCAT training system based on standardized disaster‐relief protocols. This training has become a prerequisite for PCAT registration, ensuring higher‐quality disaster response and preparedness [34].

### 8.2 Infectious Disease Countermeasures

During the COVID‐19 pandemic, which spread globally from late 2019, the JPCA's Infectious Disease Committee played a pivotal role in disseminating information and conducting educational activities in collaboration with other academic societies and the Japanese government. The committee's Vaccine Team and Infectious Disease Treatment and Countermeasures Team guided prevention and treatment strategies [35].

In addition, the JPCA has been actively involved in vaccine education across all age groups. One of its key initiatives is the “Vaccination for All” (*Kodomo to Otona no Vaccine*) website, an online platform that provides cross‐generational vaccine information to improve public awareness and promote the prevention of infectious diseases [36].

### 8.3 The Hinohara Award and the Tasaka Award

The former Japanese Society of General Medicine established the Hinohara Award in 2001 to honor Dr. Shigeaki Hinohara, former director of the St. Luke's International Hospital, for his significant contributions to primary care in Japan. The award also aims to support and nurture young researchers in this field. The JPCA has presented the Hinohara Award since the merger, and a total of 36 recipients (including 19 since the JPCA's formation) have received the honor, as of 2024 [37].

In addition to the Hinohara Award, the JPCA provides research funding and mentorship to support a wide range of academic projects. In 2017, the association launched the Future Research Leader Development Program to provide mentorship and financial support to young researchers. This initiative has contributed to numerous academic publications and advancements in primary care research [38].

The Tasaka Award, established by the Japanese Academy of Family Medicine in 2008, honors Dr. Yoshikazu Tasaka, a pioneer in family medicine in Japan, who passed away unexpectedly in 2007. The award recognizes individuals who have made outstanding contributions to family medicine education, dissemination, and quality improvements. The JPCA has continued this tradition, and as of 2025, 19 individuals have been awarded the honor [39].

### 8.4 Inter‐Professional Training

Recognizing the importance of inter‐professional collaboration in primary care, the JPCA has expanded its educational initiatives beyond physicians to include nurses and pharmacists. As of 2024, the JPCA included 575 nurses and 823 pharmacists among its members.

The Japanese Medical Society of Primary Care, the predecessor of the JPCA, launched a Primary Care Pharmacist Certification Program in 2009 [40]. The program continued and expanded after the establishment of the JPCA. On February 21, 2011, the JPCA became the first medical association to receive official recognition as a “specialized area certification system” from the Council on Pharmacists Credentials, an independent body overseeing pharmacist certification in Japan [41]. This certification is more advanced and specialized than the general pharmacist training programs. As of December 2024, 263 pharmacists have been certified.

Similarly, in 2019, the JPCA launched the Primary Care Certified Nurse System to expand the role of nurses in primary care and strengthen their clinical skills [42]. The program has grown steadily with workshops, study groups, and networking events held nationwide. As of December 2024, 111 nurses have been certified under this system.

In 2020, the JPCA launched a new certification system for JPCA Certified Family Physicians. This initiative was introduced in response to the launch of the JMSB's *Sogo‐Shinryo‐Senmon‐I* as a basic specialty area, thereby positioning the JPCA certification system within the subspecialty framework [43]. Furthermore, in 2024, the system received international accreditation from WONCA [44], thereby further enhancing its international value and recognition.

## 9 Conclusion: The Present and Future of the JPCA

The JPCA has inherited the legacy of its three predecessor societies and has played a vital role in advancing primary care in Japan. Since its establishment, the JPCA has made significant contributions to the field through academic conferences, training programs, academic journal publications, physician and specialist certifications, and inter‐professional education initiatives.

Fifteen years after its founding, the JPCA continues to strengthen inter‐professional collaboration with nurses, pharmacists, dentists, caregivers, welfare workers, and other healthcare professionals. This initiative addresses Japan's demographic challenges, particularly the issues relevant to 2025, characterized by a declining birthrate and an aging population. By fostering coordinated healthcare, nursing care, and welfare systems, the JPCA promotes comprehensive primary care that meets the evolving needs of local communities.

The association also actively engages in policy discussions regarding Kakaritsuke‐I Kino Hokoku Seido (the Primary Care Physician Functioning Report System) [45], which was launched in April 2025. Through close collaboration with the government and the Japan Medical Association, the JPCA contributes to designing systems and frameworks for physician training. In addition to continuing the development of specialists such as family physicians and certified primary care physicians, the JPCA aims to strengthen its engagement with academia—both in Japan and internationally. Enhancing its presence in academic research will be fundamental to expanding its influence as a leading organization in primary health care.

The JPCA is committed to improving the health and well‐being of patients, residents, and local communities. By promoting innovative primary care models applicable to diverse regions in Japan and internationally, the JPCA aims to contribute to the global development of primary care. As it deepens its collaborations with domestic and international organizations, the association is expected to continue to play a pivotal role in shaping the future of primary care across generations.

## Author Contributions

S.M. and M.A. conceptualized and drafted the manuscript. All authors reviewed and approved the final version of the manuscript for publication.

## Ethics Statement

The authors have nothing to report.

## Consent

The authors have nothing to report.

## Conflicts of Interest

The authors declare no conflicts of interest.

## Data Availability

The authors have nothing to report.
